# Optimal dose of pretreated-dexmedetomidine in fentanyl-induced cough suppression: a prospective randomized controlled trial

**DOI:** 10.1186/s12871-019-0765-z

**Published:** 2019-06-01

**Authors:** Wei Zhou, Dongsheng Zhang, Shunping Tian, Yang Yang, Zhi Xing, Rongrong Ma, Tianqi Zhou, Tianxiu Bao, Jianhong Sun, Zhuan Zhang

**Affiliations:** 1 School of Medicine, Yangzhou University, Yangzhou, 225009 China; 2 Department of Anesthesiology, The Affiliated Hospital of Yangzhou University, Yangzhou, 225012 China; 3 Preventive Health Care Office, The Affiliated Hospital of Yangzhou University, Yangzhou, 225012 China

**Keywords:** Dexmedetomidine, Fentanyl, Cough

## Abstract

**Background:**

To investigate the optimal dose of pretreated-dexmedetomidine in fentanyl-induced cough (FIC) suppression.

**Methods:**

Patients of 180 undergoing elective surgery with general anesthesia, aged 18–65 years, BMI 18.5–30 kg/m^2^, ASA I or II, were equally randomized into four groups (*n* = 45) to receive intravenous pretreatment of dexmedetomidine with 0 (group 1), 0.3 (group 2), 0.6 (group 3) and 0.9 (group 4) mcg/kg over 10 mins, respectively. After the pretreatment, all patients were given a 5-s intravenous injection of fentanyl 4 mcg/kg. The symptoms of irritating cough including the severity and onset time were recorded for 1 min after fentanyl injection. General anesthesia induction was completed with midazolam, propofol and cisatracurium, then endotracheal tube or laryngeal mask was inserted and connected to an anesthesia machine. MAP, HR and SpO_2_ at the beginning of pretreatment (T0), 3 min (T1), 6 min (T2), 9 min (T3) and 12 min (T4) after the beginning of pretreatment were recorded. Side effects of dexmedetomidine, such as bradycardia, hypertension, hypotension, and respiratory depression were also recorded during the course.

**Results:**

Totally 168 patients completed the study. The incidences of cough were 52.4, 42.9, 11.9, and 14.3% in groups 1, 2, 3, and 4, respectively, with no significant differences between groups 1 and 2 (*P* > 0.05) and between groups 3 and 4 (*P* > 0.05). The incidence and severity of cough in groups 3 and 4 were significantly lower than those in groups 1 and 2 (*P* < 0.05). Compared to T0, HR at T2 (*P* < 0.05), T3 (*P* < 0.01), and T4 (*P* < 0.01) decreased significantly and MAP at T4 decreased significantly (*P* < 0.05) in group 4. Bradycardia occurred in 1 case and respiratory depression occurred in 1 case in group 4. Compared to group 1, the onset time of cough in the other 3 groups were delayed significantly (*P* < 0.05).

**Conclusion:**

Pretreated dexmedetomidine 0.6 mcg/kg blous intravenous infusion over 10 mins could reduce FIC effectively without side effects.

**Trial registration:**

This study was registered in ClinicalTrials.gov (NCT03126422), April 13, 2017.

## Background

Fentanyl is used widely for general anesthesia induction due to its rapid onset, intensive analgesia and cardiovascular stability; however, an irritating cough may be caused after its intravenous (IV) administration [1]. The incidence of fentanyl-induced cough (FIC) can reach 80% [2]. The FIC may be transitory and limited; however, it can be explosive and detrimental especially in patients with increased intracranial, intraocular, intrathoracic, or intra-abdominal pressure [3–5]. FIC could even cause severe upper airway obstruction and aspiration pneumonia that require immediate intervention [6, 7]. A report that explosive FIC produced multiple conjunctival and periorbital petechiae has been published [8]. FIC needs immediate and effective intervention especially in patients with cerebral aneurysm, brain trauma, hernia, open eye injury, dissecting aortic aneurysm, pneumothorax or hypersensitive airway disease. Precaution of FIC in these situations is of great importance.

The mechanism of FIC has not been elucidated definitely, although various studies have been conducted to suppress or alleviate this side effect [4, 9]. A previous study has shown that intravenous clonidine could suppress FIC effectively through its α_2_-adrenoceptor agonist effect [10]. Dexmedetomidine, a highly selective α_2_-adrenoceptor agonist, is widely used for its particular virtues, such as favorable sedative and analgesic effects. It can also reduce central sympathetic outflow and stress response [11]. A previous study has shown the suppressing effect of dexmedetomidine combined with midazolam on FIC [12]. While another study reported that better cough suppression was found at 1 mcg/kg vs. 0.5 mcg/kg dexmedetomidine bolus without an increase in side effects [13]. However, we should be concerned about its antisympathetic responses with higher doses of dexmedetomidine. Therefore, we designed a study to investigate the optimal priming dose of dexmedetomidine in FIC suppression during general anesthesia induction.

## Methods

This prospective, randomized, double-blind, controlled clinical trial was approved by the Institutional Research Ethics Committee of the Affiliated Hospital of Yangzhou University, Yangzhou, China. All the participants provided written informed consent following principles of the Helsinki Declaration. Also, this study was registered in ClinicalTrials.gov (NCT03126422).

### Participants

One hundred and eighty patients, ASA I or II, aged 18–65 years, BMI 18.5–30 kg/m^2^, and scheduled for elective surgeries under general anesthesia between Oct 2017 and May 2018, were enrolled in the study. Exclusion criteria were patients with bradycardia (HR < 50 beats/min [14]), hypotension (blood pressure < 90/60 mmHg), impairment of liver or kidney, smoking, asthma, chronic cough, upper respiratory tract infection within the previous 2 weeks, or use of medications that could interfere with this study such as angiotensin-converting enzyme inhibitors, bronchodilators, or steroids.

### Study protocol

This study was randomly assigned to four groups with 45 patients each depending on the 10-min pretreated dose of dexmedetomidine, using computer-generated random numbers: group 1 (0 mcg/kg), group 2 (0.3 mcg/kg), group 3 (0.6 mcg/kg) and group 4 (0.9 mcg/kg).

No premedication was used in all patients. Venous access was established on the wrist cephalic vein of the nondominant hand with a 20-G intravenous cannula after patients came into the pre-operation room and Ringers’ solution of 8 ml•kg^− 1^•h^− 1^ was transfused. The vertical distance from the drip bottle to the venous access was 80 cm in all the cases in this study. The IV cannula was connected to T-connectors for drugs infusion and injection in the operating theater. All patients were monitored with electrocardiogram, noninvasive blood pressure, and SpO_2_ during the whole study.

Anesthesia induction was standardized and the procedure consisted of the following. Dexmedetomidine (200 mcg/2 ml; 181016BP, Hengrui Co., Jiangsu, China) was diluted with normal saline to a concentration of 4 mcg/ml. Patients were given dexmedetomidine by pumping at an intravenous dose of 0, 0.3, 0.6, and 0.9 mcg/kg over 10 mins in groups 1, 2, 3, and 4, respectively. In group 1, normal saline was used and the infusion rate was set at 50 ml/h. All the pretreatments were prepared and implemented by an experienced anesthesiologist who was not involved in data collection. Also, all the priming drugs and the infusion pumps were covered with a piece of sheet. Oxygen supply through facemask was given to all the patients. Assisted ventilation was supplied if SpO_2_ fell below 95% or decreased by 5% from initial value throughout the study. At 10 min after the beginning of pretreatment infusion, the pumping rate of dexmedetomidine was continued at 0.5 mcg•kg^− 1^•h^− 1^ in all the groups. Meanwhile, fentanyl (50 mcg/ml; 81D05031, Renfu Co., Hubei, China) 4 mcg/kg with the injection time of 5 s was given to all the patients. A stopwatch was used to control the time.

After fentanyl injection, the symptoms of irritating cough including the severity and onset time (the time from the end of fentanyl injection to the beginning of coughing) of cough were recorded for 1 min. Any occurrence of cough was identified as coughing. According to the number of coughs within 1 min after fentanyl injection [9], the severity of cough was classified to four grades: 0 (no cough), 1 (mild, 1–2 times), 2 (moderate, 3–5 times), and 3 (severe, > 5 times). The recording was done by an anesthesiologist who was unaware of the grouping criteria.

General anesthesia induction was continued following cough cessation or 1 min after fentanyl injection with midazolam 0.05 mg/kg, propofol 1.5–2.5 mg/kg and cisatracurium 0.2 mg/kg to facilitate endotracheal intubation or laryngeal mask insertion. Mechanical ventilation was controlled with tidal volume of 8 ml/kg, at a respiratory rate of 12 breaths/min. The beginning of pretreated-dexmedetomidine use was recorded at 0 min (T0). MAP, HR, and SpO_2_ were recorded at T0, 3 min (T1), 6 min (T2), 9 min (T3) and 12 min (T4) after the beginning of pretreatment. Side effects of dexmedetomidine, such as bradycardia, hypertension, hypotension, and respiratory depression were recorded during the course. Ephedrine was used if MAP < 60 mmHg or the decrease of MAP > 30% of the basal data. Atropine was used if HR < 50 beats/min or the decrease of HR > 30% of the basal data. The relevant measures taken to deal with the side effects were also recorded. The above recordings were done by another anesthesiologist who was unaware of the grouping criteria.

### Sample size determination

In our preliminary study, the incidence of FIC was 48%. A power analysis was performed using the incidence of FIC as the primary variable. We hypothesized that certain dose of 10-min dexmedetomidine priming infusion could reduce the incidence of FIC to 15%. To detect this deference with 90% power at a 5% significance level, 40 patients would be necessary in each group. Therefore, we recruited 45 patients for each group to allow missing data.

### Statistical analysis

Statistical analysis was performed using Statistical Product for Social Sciences (SPSS) software 19.0 for windows. Data were expressed as mean ± SD, number, proportion, or percentage. Quantitative variables were analyzed using one-way ANOVA with repeated measures between groups. One-way ANOVA and post Hoc Bonferroni multiple comparison test were used to compare differences of vital signs between groups after dexmedetomidine infusion and fentanyl injection. Ordinal data were compared with the Kruskal-Wallis test followed, when indicated, with Dunn’s multiple comparison tests. *P* value of < 0.05 was considered statistically significant.

## Results

### Study subjects

**Fig. 1:**
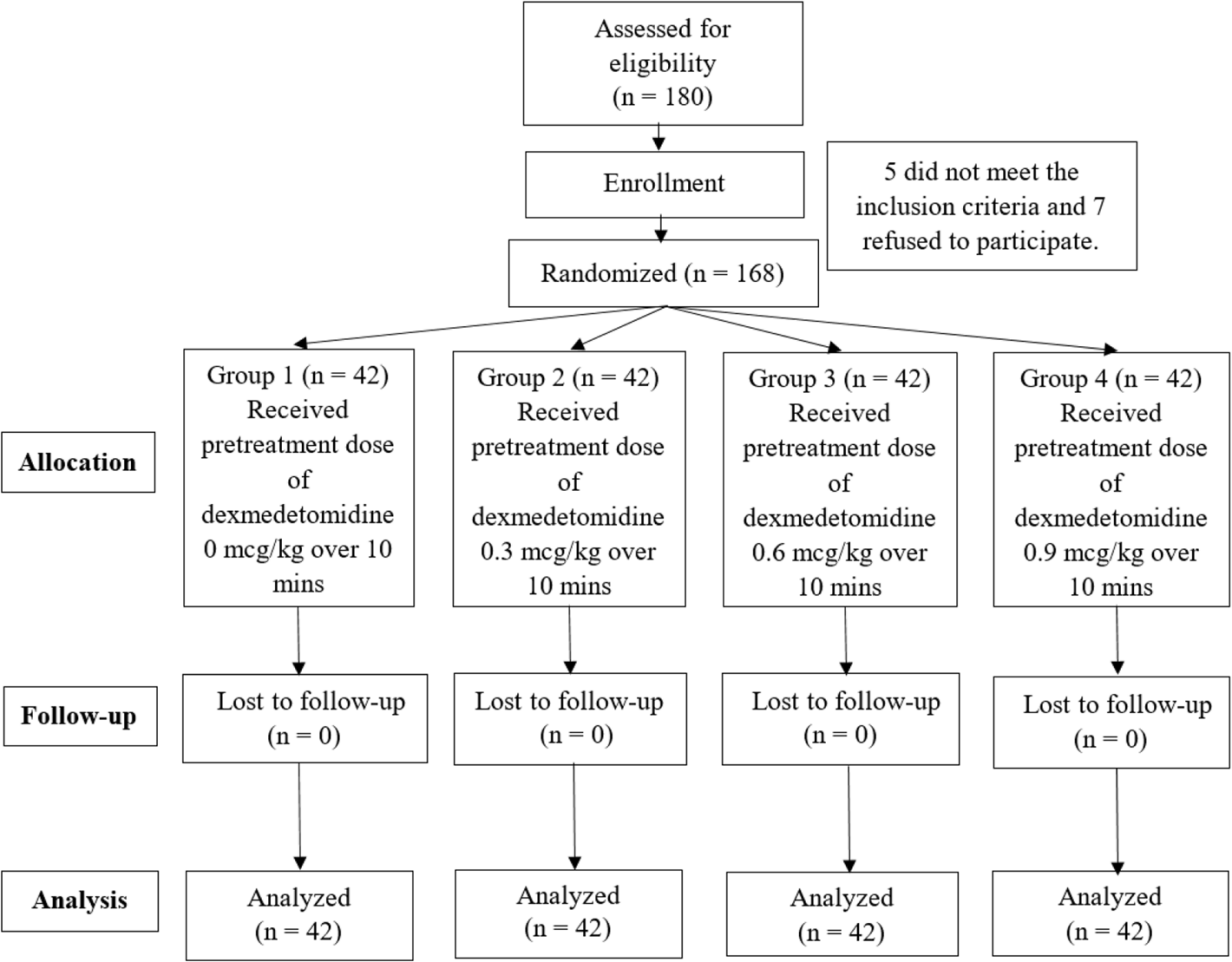
Consolidated Standards of Reporting Trials (CONSORT) recommended description of patient recruitment

In total, 180 patients were surveyed for their eligibility. Of these patients, 5 did not meet the inclusion criteria, and 7 refused to participate. The remaining 168 patients were randomized into four groups (*n* = 42) and completed the study (Fig. 1). There were no significant differences among the four groups with respect to demographic data including age, sex, BMI, and ASA physical status (*P* > 0.05) (Table 1).

**Table 1:** Demographic characteristics of patients in the four groups

Parameters	Group 1	Group 2	Group 3	Group 4
Age (years)	46.8 ± 14.3	49.9 ± 11.8	47.3 ± 13.4	45.3 ± 10.0
Gender (males/females)	19/23	20/22	18/24	21/21
Weight (kg)	68.6 ± 11.8	69.8 ± 10.6	65.7 ± 12.6	63.4 ± 9.8
BMI (kg/cm^2^)	24.5 ± 2.8	25.1 ± 3.2	24.2 ± 3.6	23.0 ± 2.3
ASA (I/II)	24/18	26/16	26/16	25/17

Values are mean ± standard deviation

### Effects of pretreatments on incidence and severity of cough

There were 22 (52.4%), 18 (42.9%), 5 (11.9%), and 6 (14.3%) patients had coughs in groups 1, 2, 3, and 4, respectively. No significant differences between groups 1 and 2 and between groups 3 and 4 were found (*P* > 0.05). Compared to groups 1 and 2, the incidence of cough in groups 3 and 4 decreased significantly (*P* < 0.05). (Table 2).

**Table 2:** Onset time, incidence and severity of cough in the four groups

Cough		Group 1	Group 2	Group 3^ac^	Group 4^ac^
Onset time (s, ^−^*x* ± *s*)		11.8 ± 4.5	17.5 ± 6.5^b^	17.4 ± 5.7^b^	17.2 ± 5.8^b^
Incidence (n, %)		22 (52.4)	18 (42.9)	5 (11.9)	6 (14.3)
Severity (n, %)	0 (No cough)	20 (47.6)	24 (57.1)	37 (88.1)	36 (85.7)
	1 (Mild)	8 (19.0)	8 (19.0)	3 (7.1)	5 (11.9)
	2 (Moderate)	8 (19.0)	6 (14.3)	1 (2.4)	1 (2.4)
	3 (Severe)	6 (14.3)	4 (9.5)	1 (2.4)	0 (0)

Onset time: from the end of fentanyl injection to the beginning of coughing

^a^ *P* < 0.01, ^b^ *P* < 0.05 compared to group 1, ^c^ *P* < 0.05 compared to group 2

The severity of cough in the four groups was shown in Table 2. There were no significant differences about it between groups 1 and 2 and between groups 3 and 4 (*P* > 0.05). The severity of cough decreased significantly in groups 3 and 4 compared to groups 1 and 2 (*P* < 0.05).

### Effects of pretreatments on the onset time of cough

The onset time of cough was 11.8 ± 4.5 s, 17.5 ± 6.5 s, 17.4 ± 5.7 s, and 17.2 ± 5.8 s in groups 1, 2, 3, and 4, respectively (Table 2). Compared to group 1, the pretreatment of dexmedetomidine delayed FIC onset time significantly in groups 2, 3, and 4 (*P* < 0.05). However, there were no significant differences about it between groups 2, 3, and 4.

### Safety

**Fig. 2:**
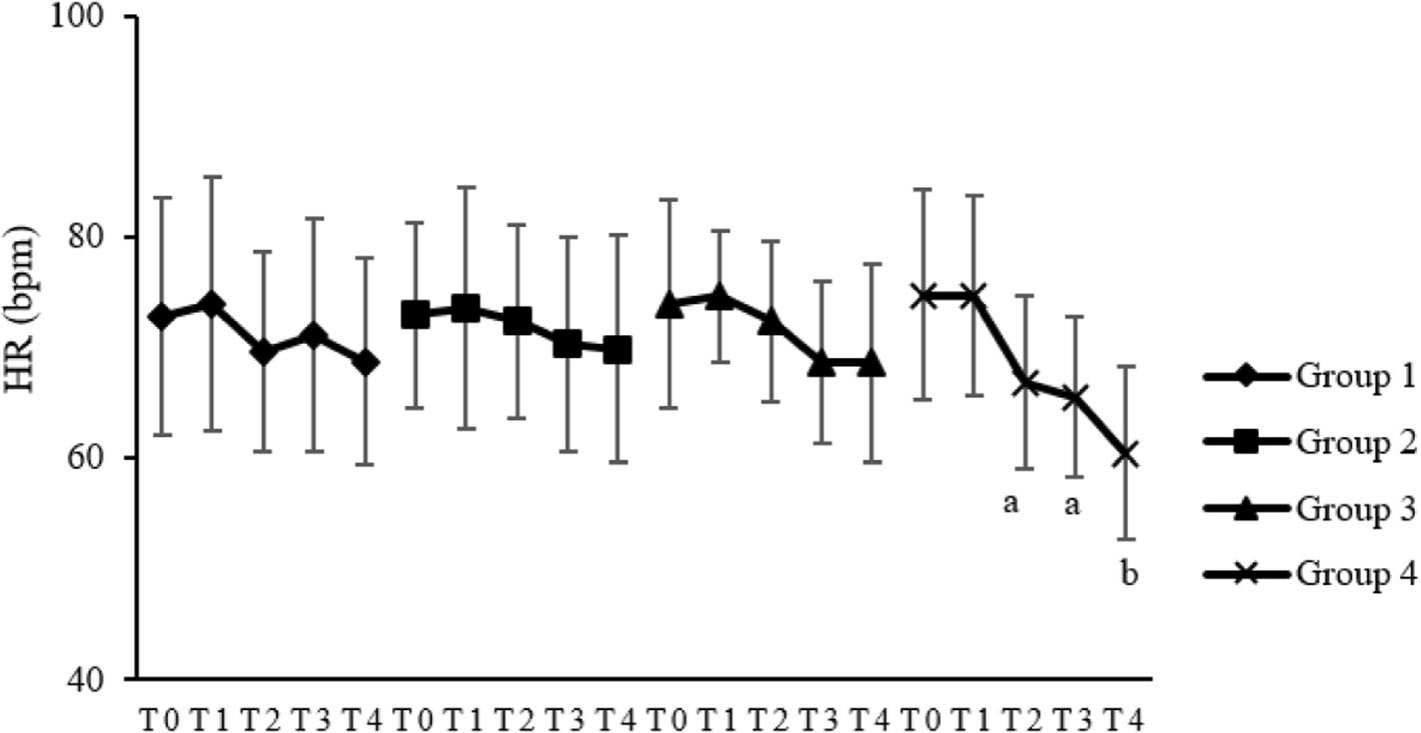
Effects of pretreatments on HR in the four groups. HR: heart rate; bpm: beats/min. T0: at the beginning of pretreatment; T1: 3 min after the beginning of pretreatment; T2: 6 min after the beginning of pretreatment; T3: 9 min after the beginning of pretreatment; T4: 12 min after the beginning of pretreatment. ^a^ *P* < 0.05, ^b^ *P* < 0.01, compared to T0

**Fig. 3:**
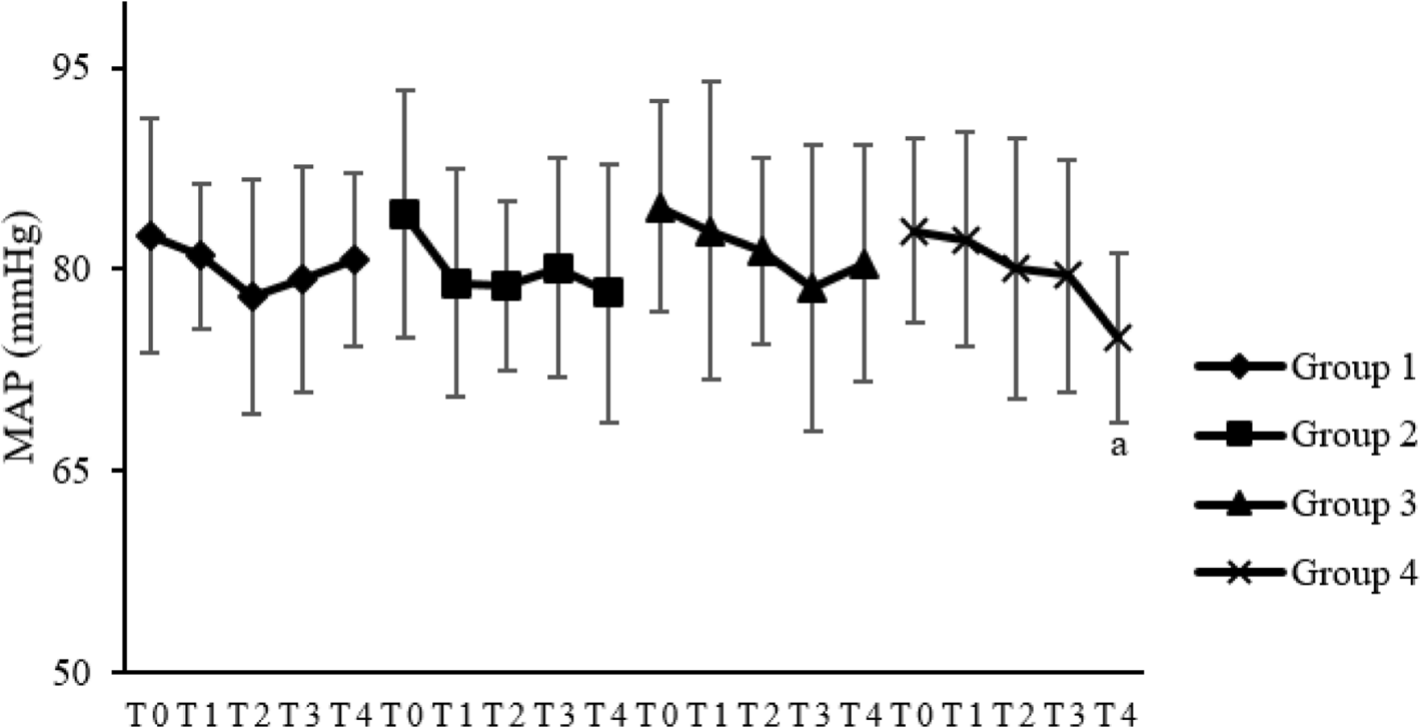
Effects of pretreatments on MAP in the four groups. MAP: mean arterial pressure. T0: at the beginning of pretreatment; T1: 3 min after the beginning of pretreatment; T2: 6 min after the beginning of pretreatment; T3: 9 min after the beginning of pretreatment; T4: 12 min after the beginning of pretreatment. ^a^ *P* < 0.05, compared to T0

There were no significant differences in MAP and HR between groups 1, 2, and 3. Compared to T0, HR and MAP in groups 1, 2 and 3 were not significantly different at the other three time points (*P* > 0.05). In group 4, HR at T2 (*P* < 0.05), T3 (*P* < 0.05) and T4 (*P* < 0.01) decreased significantly compared to T0 (Fig. 2); MAP at T4 decreased significantly compared to T0 (*P* < 0.01) (Fig. 3). No serious adverse events occurred during the study. No bradycardia, hypertension, hypotension, or respiratory depression occurred in groups 1, 2, and 3. While in group 4, one patient developed bradycardia and needed atropine treatment, and 1 patient had respiratory depression with SpO_2_ < 95% and assisted ventilation was effective.

## Discussion

The present study discovered that pretreatment with dexmedetomidine intravenous infusion of 0.6 mcg/kg bolus given over 10 mins reduced the severity of FIC effectively without adverse effects when fentanyl 4 mcg/kg was injected with the injection time of 5 s.

FIC deserves enough attention. Previous studies have shown that the incidence of FIC is about 35–64% [1, 5, 15, 16].These discrepancies may be due to differences in fentanyl injection dose, speeds, or routs. In this study, we used fentanyl 4 mcg/kg as the dose was usually adopted for general anesthesia induction in our daily work. We found that 52.4% patients had cough in the control group when fentanyl was injected through the wrist cephalic vein. A higher incidence of cough occurred in our control group than in some previous reports [4, 17], which was probably due to the rapid injection of fentanyl bolus (5 s of 4 mcg/kg) in our study. In clinical practice, fentanyl might be injected more slowly. Besides, there were more females in our study. Females are susceptible to FIC [18].

The mechanism of FIC has not been fully elucidated. However, a number of possible theories have been proposed: (1) The trigger stimulus and bronchial hyper-irritability theory might be a reason. Opioid receptors have been identified in the trachea, bronchi, and alveolar walls. The opioid receptors can be activated by fentanyl and airway smooth muscles can be triggered to constrict. Histamine and neuropeptides may be released by action on the prejunctional μ-opioid receptors after fentanyl injection. Irritating-cough then is produced [8, 19]; (2) A pulmonary chemoreflex is another likely mechanism, which is mediated by either irritant receptors or by vagal C-fiber receptors near pulmonary vessels [20]; (3) Muscle rigidity caused by fentanyl might induce sudden adduction of the vocal cords or supraglottic obstruction and cough might happen [20]; (4) The balance between sympathetic nerve and parasympathetic nerve may also have an effect on FIC [21]. The highly selective α_2_-adrenergic agonist dexmedetomidine, with sedative and analgesic properties, is mostly used in clinical applications. Due to the central nervous system effect of fentanyl, we speculate that the suppression of FIC by preemptive infusion of dexmedetomidine might also be related to the fact that it could penetrate into blood brain barrier and suppress cough reflex by inhibiting the cough center directly due to its high lipid solubility [20].

Medications or mechanical measures have been used to relieve FIC [2, 21–23]. However, the effects varied from each other. In Liang H et al’s study, they found that intravenous dexmedetomidine (0.5 mcg/kg or 1 mcg/kg) immediately before fentanyl (4.0 mcg/kg) injection reduced the incidence of FIC [13]. The risk of bradycardia is significantly higher when the loading dose is greater than 0.7 mcg/kg [24]. It is practical to explore the applicability of different doses of dexmedetomidine and find out the optimal dose of pretreated-dexmedetomidine with effective suppression on FIC and without side effects. In clinical practice, loading dose was often infused over 10 mins and a maintenance dose was then continued [25]. In the present study, dexmedetomidine was infused over 10 mins to achieve the steady plasma concentration.

In this study, we applied different doses of pretreated-dexmedetomidine intravenously to explore the optimal dose of dexmedetomidine in suppressing FIC. This study demonstrated that intravenous pretreatment of dexmedetomidine 0.6 mcg/kg bolus given over 10 mins could effectively decrease the incidence and severity of FIC. As was seen in group 4 in our study, compared to T0, MAP at T4 decreased significantly and HR at T2, T3, and T4 decreased significantly in group 4. This may be due to the exciting effects of α_2_-adrenergic receptors of dexmedetomidine and the according decreased release of catecholamine. After pretreatment in group 4, HR decreased to below 50 beats/min in 1 patient and was treated with atropine effectively. SpO_2_ was seen decreasing to below 95% in 1 case and increased to 100% after pressurized auxiliary ventilation during dexmedetomidine infusion also in group 4. The onset time of FIC in group 3 was about 17.4 s later after fentanyl injection in this study. The peak plasma concentration of fentanyl in lung parenchyma could decrease over time because of its high lipid solubility and the absorption of other tissues. A low plasma concentration of fentanyl might not induce FIC. Prolonging the injection time of fentanyl over the time to reach the threshold of its plasma concentration could reduce the incidence and severity of FIC further.

There are still some limitations in this study. First, the judgment index of FIC incidence and degree is subjective, for no objective indicators have been found till now. Second, this study focused on clinical manifestation of the inhibitory effect of dexmedetomidine on FIC, while the specific physiological mechanism needs further study.

## Conclusion

In conclusion, the pretreated intravenous infusion of dexmedetomidine 0.6 mcg/kg bolus given over 10 mins before a 5-s injection of fentanyl 4 mcg/kg can suppress FIC effectively without side effects.

## Data Availability

The datasets used or analyzed during the current study are available from the corresponding author on reasonable request.

## Abbreviations

BMI: Body mass index
FIC: Fentanyl-induced cough
HR: Heart rate
MAP: Mean arterial pressure
SpO_2_: Pulse oxygen saturation
